# Viral community succession during cadaver decomposition and its potential for estimating postmortem intervals

**DOI:** 10.1128/aem.01453-25

**Published:** 2025-09-16

**Authors:** Daijing Yu, Yaru Mai, Liuyaoxing Zhang, Yuntian Xiao, Meng Zhang, Bo Shao, Boxian Chen, Tian Wang, Kewen Zhang, Liwei Zhang, Niu Gao, Jun Zhang, Jiangwei Yan

**Affiliations:** 1 School of Forensic Medicine, Shanxi Medical University 74648, Jinzhong, Shanxi, China; 2 Shanxi Key Laboratory of Forensic Medicine, Jinzhong, Shanxi, China; 3 Shanxi Province Engineering Research Center of Forensic Identification, Taiyuan, Shanxi, China; 4 School of Forensic Medicine, Inner Mongolia Medical University 66287 , Hohhot, Inner Mongolia, China; 5 MOE Key Laboratory of Coal Environmental Pathogenicity and Prevention, Jinzhong, Shanxi, China; University of Nebraska-Lincoln, Lincoln, Nebraska, USA

**Keywords:** postmortem interval (PMI), viral succession, bacteriophage, machine learning, metagenomics

## Abstract

Microbial communities play a vital role in cadaver decomposition and serve as reliable tools for postmortem interval (PMI) estimation. However, current research focuses primarily on bacterial/fungal communities, though viruses—as Earth’s most abundant biological entities—play key roles in biogeochemical cycles by regulating bacterial communities via lysis-lysogeny switching. Viral succession patterns during decomposition remain insufficiently characterized, and their PMI biomarker potential is unexamined. We present metagenomic analysis of viral succession during 35-day decomposition of buried rat cadavers, revealing stage-specific dynamics: early dominance of Peduoviridae (0–3 days), mid-stage proliferation of Herelleviridae (7–21 days), and late-stage resurgence of Peduoviridae (28–35 days). Viral α-diversity exhibits a fluctuating downward trend. β-Diversity analysis (PCoA, ANOSIM, PERMANOVA; *P* < 0.001) confirmed PMI as a major structural driver (27% variance explained). Nine viral families exhibited significant PMI correlations (*P* < 0.05): Zierdtviridae, Casjensviridae, Schitoviridae, and Ackermannviridae showed strong negative correlations (*r* = −0.82 to −0.78), while Straboviridae correlated positively (*r* = 0.59). Using integrated viral abundance data, our Extremely Randomized Trees model achieved exceptional PMI prediction accuracy (test set: *R*² = 0.96, MAE = 2.54 days). Spearman correlations between dominant bacterial phyla (Bacteroidota, Bacillota, etc.) and viral families, combined with Procrustes analysis (*M*² =0.3385, *P* = 0.001) and Mantel tests (*r* = 0.8059, *P* = 0.001), confirmed strong virus-bacteria community consistency. This indicates viruses may regulate decomposition by targeting bacteria for lysis, releasing nutrients (e.g., organic nitrogen/phosphorus) to drive bacterial succession. Our study establishes a novel virus-based PMI prediction tool and discusses ecological drivers of decomposition.

**IMPORTANCE:**

We present a viral succession-based framework for estimating PMI in buried remains. Our study identifies stage-specific viral biomarkers and identified nine viral families significantly correlated with PMI. By combining metagenomics and machine learning, we developed an Extremely Randomized Trees (ERT) model that achieved a low prediction error (test set: *R*² = 0.96, MAE = 2.54 days). Furthermore, our findings demonstrate that viral and bacterial communities exhibit significant consistency and correlation during cadaver decomposition. This study not only provides a novel tool for the accurate estimation of forensic PMI but also advances our insight into viral regulation of bacteria and their interactions during cadaver decomposition.

## INTRODUCTION

Cadaver decomposition is an indispensable process in ecosystems, playing a vital role in energy flow, nutrient cycling, and the maintenance of biodiversity. Through the activities of microorganisms, invertebrates, and other decomposers, organic matter in cadavers is broken down into inorganic nutrients (such as nitrogen, phosphorus, and potassium), which re-enter the ecosystem and support plant growth and the survival of other organisms (1). Cadaver decomposition significantly alters the structure and function of microbial communities in local environments; this process creates unique ecological niches by releasing nutrients and modifying physicochemical properties (2–4). Therefore, studying cadaver decomposition not only enhances our understanding of ecosystem functions but also provides critical insights for forensic investigations (5).

Microbial communities, particularly bacteria and fungi, play a key role in cadaver decomposition. The predictable succession patterns of these communities have been extensively studied. These communities, which in the living human body outnumber human cells by approximately tenfold (6), undergo dynamic changes involving proliferation, death, and transformation of immense microbial populations (7). These predictable changes, collectively referred to as the thanatomicrobiome, have been leveraged as a “microbial clock” for estimating the postmortem interval (PMI), defined as the time elapsed since death (8).

Recent research has demonstrated the utility of microbial succession in PMI estimation for both animal and human cadavers. For example, Metcalf et al. (9) used high-throughput sequencing to study microbial succession in decomposing mouse cadavers, revealing consistent and reproducible changes in cadaveric microbial communities over time. Their findings were later extended to human cadavers, further validating the use of microbial succession for PMI estimation (10). Similarly, Liu et al. (11) applied artificial neural networks with microbial community data to achieve highly accurate PMI estimates. Fungal succession has also been explored as a complementary tool to bacterial-based methods, demonstrating its potential for PMI estimation in diverse environments (12). Advancements in machine-learning algorithms have further enhanced the accuracy and applicability of microbiome-based PMI estimation. For instance, random forest algorithms have been successfully applied to human intestinal microbiome data, enabling precise PMI predictions even in complex environmental scenarios (13, 14). These developments highlight the growing importance of microbial ecology in forensic science and offer a reliable and scientifically robust approach to PMI estimation.

As one of the most abundant biological entities on Earth (15, 16), viruses are indispensable components of decomposition ecosystems. Mounting evidence indicates that they play a critical role in driving microbial community functions and ecological processes, providing insights unattainable through bacterial studies alone by bacterial community studies alone. Bacteriophages (viruses that specifically infect bacteria) are not merely passive participants in decomposition; rather, through highly specific interactions with their hosts, they act as active regulators (17), capable of precisely targeting and modulating key bacterial populations (e.g., dominant bacterial hosts) (18). Crucially, their lytic activity serves as a “top-down” control mechanism, releasing intracellular nutrients via cell lysis (the “viral shunt”) (19), thereby directly promoting nutrient cycling and shaping microbial community structure. Meanwhile, a unique feature of viruses is lysogeny, in which viral genomes integrate into the host genome. This process introduces auxiliary metabolic genes (AMGs), which can modify host metabolism (e.g., carbon/nitrogen cycling) and facilitate horizontal gene transfer, thereby enhancing host adaptability (20–23). AMGs constitute functional modules a class of functional modules introduced by viruses, which may be absent or inactive in the core microbiome. Moreover, the dynamic balance between lytic and lysogenic cycles, as a virus-specific response mechanism, modulates the stability and diversity of microbial communities. Thus, in-depth study of viral communities in decomposition ecosystems is crucial for revealing their role in driving bacterial community succession, nutrient cycling, and maintaining biodiversity.

Although bacterial communities have been extensively studied for forensic identification, exploration of viral applications—particularly those involving the virome—remains relatively limited. Preliminary studies highlight the forensic utility of viruses: Ikegaya et al. leveraged JC polyomavirus for geographical sourcing (24); Wilson et al. outlined protocols for viral evidence analysis (e.g., viral weaponization, transmission tracing) (25); and the HidSkinPlex system incorporates Cutibacterium phage P101A as a skin biomarker (26). Graham et al. further identified 59 stable, individualized viral markers in skin viromes (27). Compared to bacterial markers, viruses exhibit environmental stability—e.g., eukaryote-infecting viruses are less susceptible to antibiotics or antimicrobial agents, providing independent and reliable alternative markers when bacterial markers fail (27, 28). Simultaneously, they demonstrate enhanced transfer and detection characteristics: their concentrations typically exceed bacteria by 1–2 log units (29); free viral particles transfer more readily to evidentiary surfaces; and VLP enrichment technology significantly improves recovery rates in trace or degraded samples (30).

Despite the significant ecological importance and unique advantages of viruses, their community dynamics during cadaver decomposition remain poorly understood. Cadaver decomposition creates a dynamic environment characterized by drastic changes in nutrient availability, pH, and microbial activity (31, 32), which are likely to drive significant succession of viral communities. However, the specific succession patterns of viruses during this process and their potential as biomarkers for PMI estimation have not been systematically studied. The advancement of metagenomic sequencing technology provides reliable technical support for comprehensively characterizing viral communities in decomposition environments and exploring the relationship between viral succession and PMI in depth.

In this study, we explored viral communities’ potential for PMI estimation in buried cadavers. To address viruses’ underexplored forensic and ecological roles, we developed a precise PMI estimation model that tracks viral succession in buried rat cadavers over 35 days. Using metagenomic sequencing of rectal samples, we characterized virome dynamics. We analyzed viral successional patterns, identified family-level viral biomarkers linked to PMI, and leveraged machine learning to evaluate predictive performance. Additionally, we analyzed the consistency and correlation between viral and bacterial communities during cadaver decomposition and further discussed virus-bacteria interactions in the process of decay.

## RESULTS

### The ecology of virus community changes during decomposition

**Fig 1:**
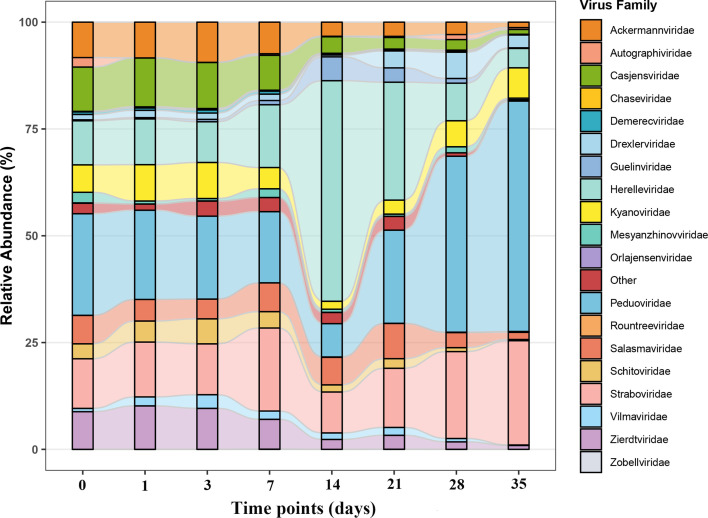
Temporal dynamics of viral family relative abundance during buried cadaver decomposition. (*X*-axis: Post-mortem interval [days]; *Y*-axis: Relative abundance [%]).

In our study, over the duration of the 35-day experiment, a total of 183,466,395 virus sequences were generated from 48 rectal samples from buried SD rat cadavers by sequencing. These sequences were clustered into 26,453 vOTUs. We investigated the virus composition of samples at eight time points at the class level (Fig. S1), and the highest relative abundance was that of Caudoviricetes. Nineteen families have been precisely classified at the family level. Viruses that could not be classified at the family level were categorized as “Other.” The viral community structure exhibited significant temporal succession during decomposition (Fig. 1). In the early stages (0–3 days), Peduoviridae occupied the dominant position (relative abundance: 23.3% at day 0 to 19.4% at day 3), followed by Straboviridae (11.7%–12.0%). Straboviridae became dominant (19.5%) at day 7, while Peduoviridae declined to 16.4%, and Herelleviridae increased significantly to 15.0%. During the mid-stage (14–21 days), Herelleviridae expanded explosively to 40.4% at day 14, becoming the dominant group; Peduoviridae (10.4% at day 14) and Straboviridae (12.7%) occupied secondary positions. By day 21, Herelleviridae maintained its abundance advantage at 28.1%, while Peduoviridae rebounded to 18.3%. In the late stage (28–35 days), Peduoviridae regained dominance (39.8% at day 28 to 50.5% at day 35, reaching peak abundance), with Straboviridae also synchronously increasing to 24.2%, whereas Herelleviridae progressively declined to 6.2%. Concurrently, Casjensviridae (declining from 10.1% to 1.3%), and Zierdtviridae (declining from 9.3% to 1.3%) demonstrated sustained decline, and Ackermannviridae peaked at day 3 (9.5%) followed by continuous decrease (1.7% at 35 day).

### The α and β diversity analysis of the viral community during cadaver decomposition

**Fig 2:**
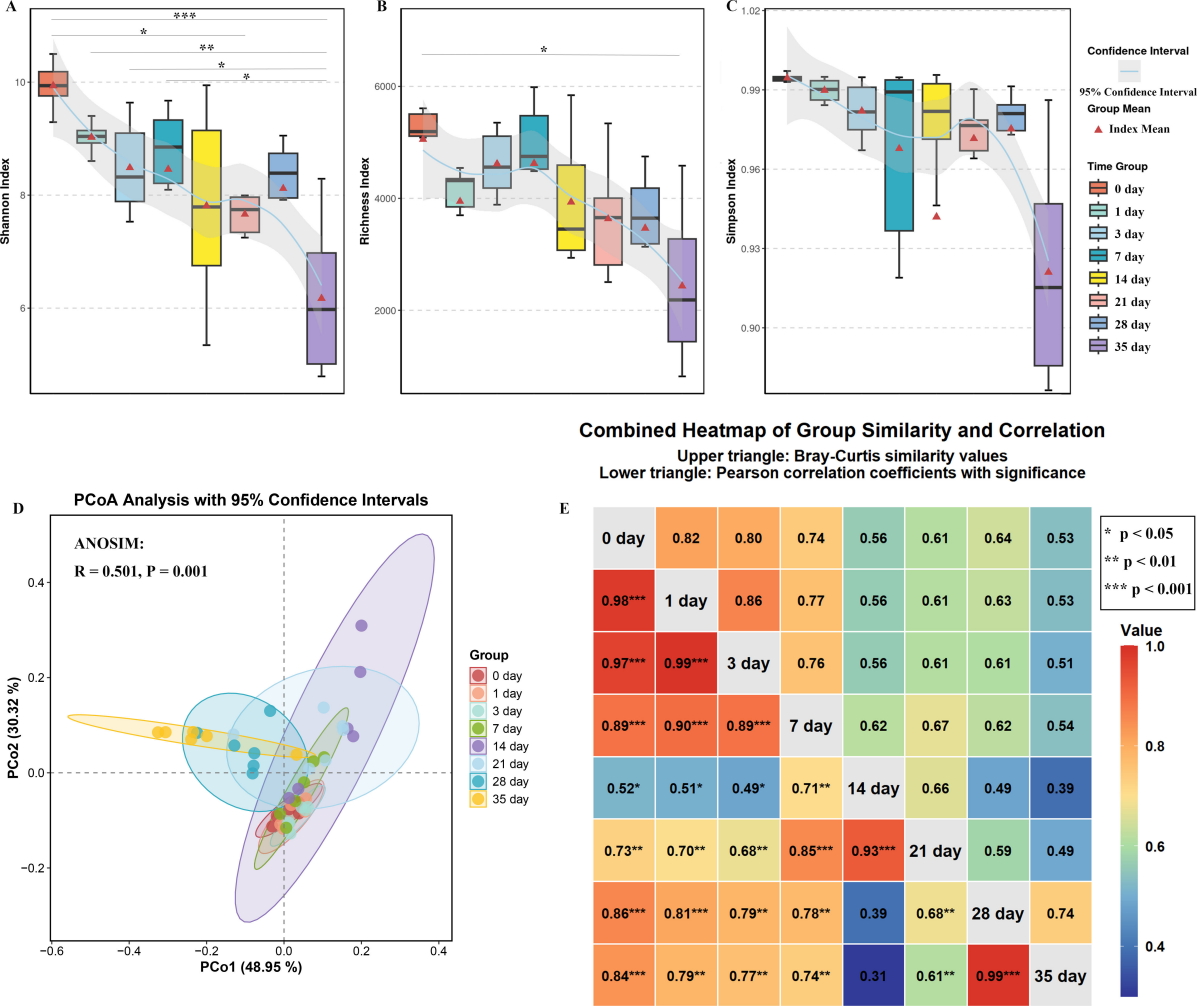
Viral community diversity during buried cadaver decomposition. Alpha diversity indices at different time points: (**A**) Shannon index, (**B**) Richness index, (**C**) Simpson index. (**D**) Beta diversity: Principal Coordinates Analysis (PCoA) based on Bray-Curtis distances showing temporal patterns (ANOSIM *R* = 0.501, *P* = 0.001). (**E**) Community similarity and temporal correlation: Upper triangle: Pairwise community similarity (1—Bray-Curtis distance). Lower triangle: Pearson correlation coefficients between decomposition stages.

Alpha (α) diversity analysis assessed viral diversity across various PMIs using the Shannon index (community diversity), Richness index (species richness), and Simpson index (community evenness or dominance). All indices showed a fluctuating decline throughout decomposition (Fig. 2A through C), indicating reduced viral diversity. The 95% confidence intervals depicted dispersion across time points, while the average value of the diversity index represented central tendencies. Significant differences were observed (e.g., Shannon index: Day 0 vs Day 35, *P* < 0.001), further supporting the divergence between early-stage (0–3 days) and mid-late-stage viral communities (14–35 days).

Principal coordinates analysis (PCoA) based on Bray-Curtis distances visualized beta (β) diversity (community structure differences) in two-dimensional space (Fig. 2D). The first axis (PCo1, 48.95% of variance) primarily differentiated communities by PMI, while the second axis (PCo2) accounted for an additional 30.32% of variance. The 95% confidence ellipses showed temporal variation patterns: high overlap among early-stage groups (0–7 days) indicated structural homogeneity, whereas mid-late-stage groups (14–35 days) exhibited separation. ANOSIM results (*R* = 0.501, *P* = 0.001) and permutational multivariate analysis of variance (PERMANOVA) collectively confirmed that PMI serves as a significant factor influencing viral community structure (Table S1), accounting for approximately 27% of variance explained by *R*² =0.27, *P* < 0.001.

Community dynamics were visualized using a heatmap combining upper-triangle similarities (1—Bray-Curtis) and lower-triangle Pearson correlations (Fig. 2E), revealing three distinct succession stages. During the early stage (0–3 days), high compositional similarity (0.80–0.86) and strong correlations (*r* = 0.97-0.99, *P* < 0.001) indicated structural homogeneity. As decomposition progressed to the pronounced succession stage (14–21 days), both similarity values and correlations significantly weakened—exemplified by comparisons between Day 14 vs Day 0 comparisons showing reduced similarity (0.56) and correlation (*r* = 0.52, *P* < 0.05). Communities at this stage also diverged markedly from later time points, as evidenced by low similarity (0.39) and correlation (*r* = 0.31) between Day 14 and Day 35. Finally, in the late stabilization stage (28–35 days), while viral community structure showed maximal dissimilarity to initial stages, intra-stage coherence rebounded sharply, with comparisons between Day 35 and Day 28 exhibiting high similarity (0.74) and very strong correlation (*r* = 0.99, *P* < 0.001), indicating ecological specialization and structural stabilization.

### Correlation between virus families and PMI

**Fig 3:**
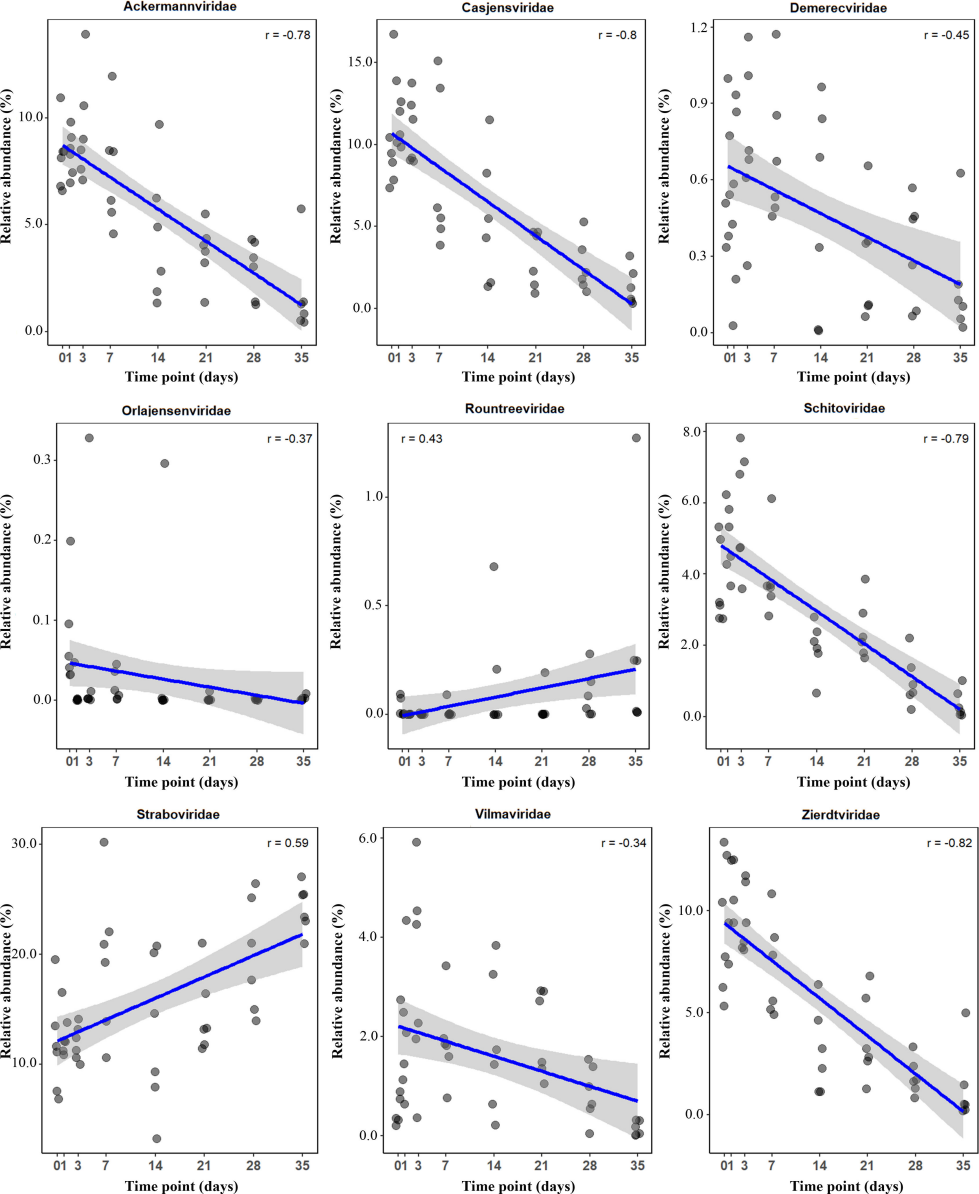
Correlation between viral family abundance and PMI. Scatter plots show abundance trends of selected viral families over time (0–35 days). Spearman correlation coefficient (*R*) indicates the relationship strength and direction.

We analyzed the correlation between each of the 19 virus families and PMI and found that 9 families exhibited significant correlations with PMI (*P* < 0.05). Among these, seven showed negative correlations, while two exhibited significant positive correlations (Fig. 3). Zierdtviridae, Casjensviridae, Schitoviridae, and Ackermannviridae all exhibited strong negative correlations, with correlation coefficients (*r*) of −0.82, −0.80, −0.79, and −0.78, respectively. In contrast, Vilmaviridae (*r* = −0.34) and Orlajensenviridae (*r* = −0.37) showed moderate negative correlations. Meanwhile, Straboviridae (*r* = 0.59) and Rountreeviridae (*r* = 0.43) showed positive correlations. The remaining 10 families did not show significant correlations with PMI (Fig. S2).

We further evaluated the inter-family correlations (Fig. S3). Significant correlations were observed between Zierdtviridae and Ackermannviridae and between Casjensviridae and Straboviridae. Moreover, we observed that Ackermannviridae showed strong correlations with both Casjensviridae and Demerecviridae.

### Predicting the PMI of burial cadavers based on virus community succession

**Fig 4:**
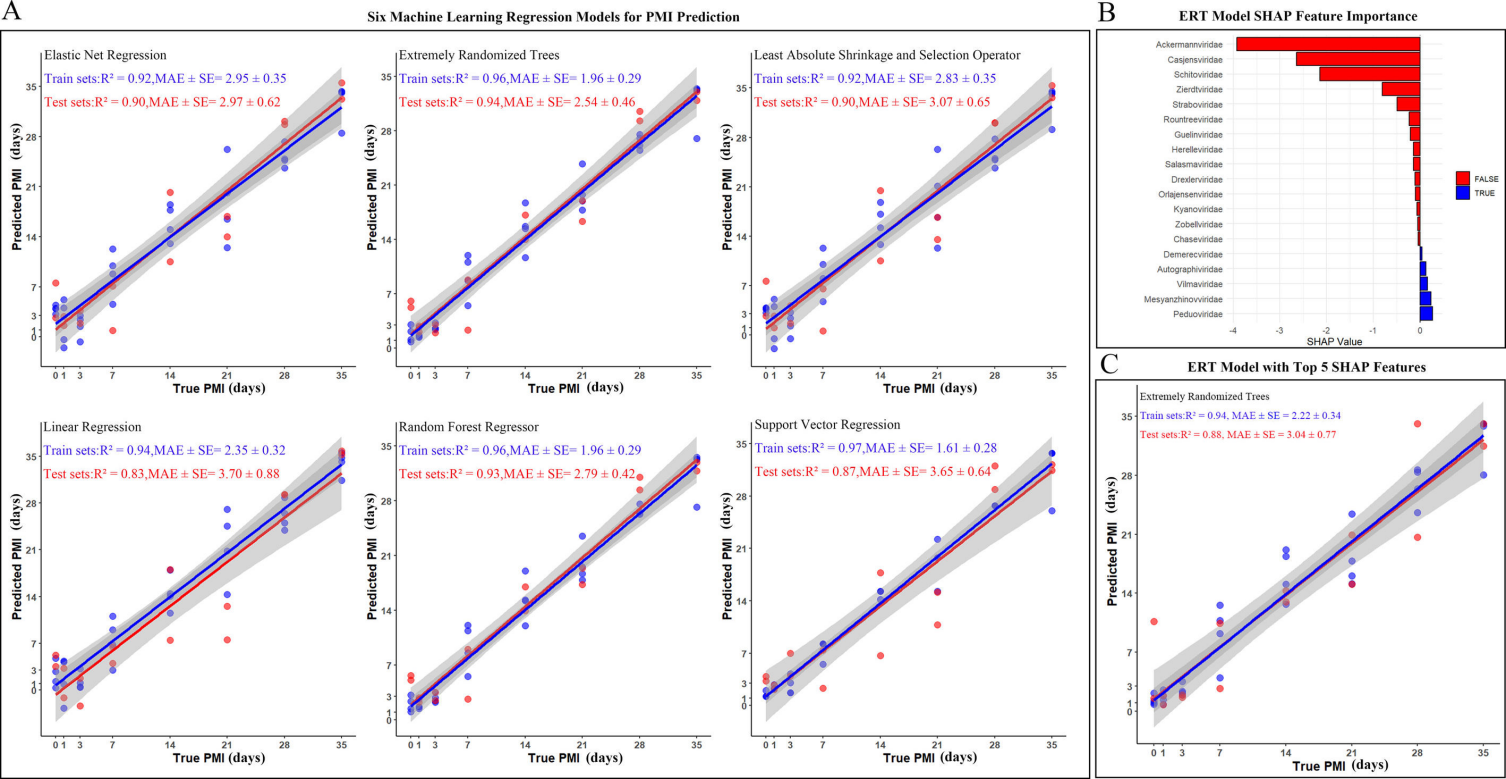
Machine-learning model performance for PMI prediction. (**A**) Predicted vs true PMI for evaluated models: Elastic Net Regression, Linear Regression, Extremely Randomized Trees (ERT), Random Forest Regressor, Least Absolute Shrinkage and Selection Operator (LASSO), Support Vector Regression. *R*² and MAE ± SE values shown for training and test sets. (**B**) ERT model features importance: SHAP values indicating the contribution of viral families. (**C**) Top-feature ERT model performance: Predicted vs true PMI using the top five viral families, with *R*² and MAE ± SE values.

We developed regression models using the relative abundance of viruses at the family level (19 families) to predict PMI, employing 6 machine-learning algorithms: Elastic Net Regression (ENR), Extremely Randomized Trees (ERT), Least Absolute Shrinkage and Selection Operator (LASSO), Linear Regression (LR), Random Forest Regression (RF), and Support Vector Regression (SVR) (Fig. 4A). During model development, training and test sets were generated using stratified random sampling by group (2:1 ratio) to maintain sample distribution across time groups. Hyperparameter optimization was conducted via fivefold cross-validation. To prevent overfitting, L1/L2 regularization penalties were applied to ENR and LASSO, while tree-based models (ERT, RF) employed feature bagging and multi-tree ensemble strategies with early stopping when applicable. Embedded feature selection utilized regularization-induced sparsity in LASSO/ENR and Gini importance evaluation in tree models, ensuring robust generalization. Results showed that the ERT model achieved the best performance (Test set: *R*² = 0.96, MAE ± SE = 2.54 ± 0.46). Its effectiveness stems from randomized split rules and a multi-tree ensemble structure that reduces variance. The RF model performed closely behind (Test set: *R*² = 0.93, MAE ± SE = 2.79 ± 0.42), benefiting from out-of-bag error estimation and feature importance screening for improved generalization. The ENR model also performed well (Test set: *R*² = 0.90, MAE ± SE = 2.97 ± 0.62) by balancing feature selection with complexity control through combined L1/L2 regularization. In contrast, the LR model had lower predictive accuracy (Test set: *R*² = 0.83, MAE ± SE = 3.70 ± 0.88) due to its linearity assumption and lack of robust regularization mechanisms.

Some virus families showed minimal or even negative impacts on the predictive accuracy of PMI. To interpret the predictions of the ERT model, we employed SHAP values, a game theory-based method for assessing feature importance that accurately measures each feature’s contribution to the model’s predictions (Fig. 4B). The results revealed significant variability in feature SHAP values among different features, with large absolute values indicating their considerable influence on the model’s predictions. Specifically, Ackermannviridae exhibited the largest absolute SHAP value, followed by Casjensviridae and Schitoviridae. The negative distribution of SHAP values indicates an inverse relationship between the changes in these viral families’ abundances and PMI; as the feature values increase, the model’s accuracy tends to improve. This suggests that Ackermannviridae, Casjensviridae, and Schitoviridae are key factors affecting the model’s predictions. Next, we identified the top five species based on their SHAP values and used them to reconstruct the models, evaluating their predictive performance. The ERT model exhibited *R*² and MAE ± SE values of 0.94 and 2.22 ± 0.34 for the training set, and 0.88 and 3.04 ± 0.77 for the test set, respectively (Fig. 4C).

### Bacterial composition and diversity dynamics during cadaver decomposition

**Fig 5:**
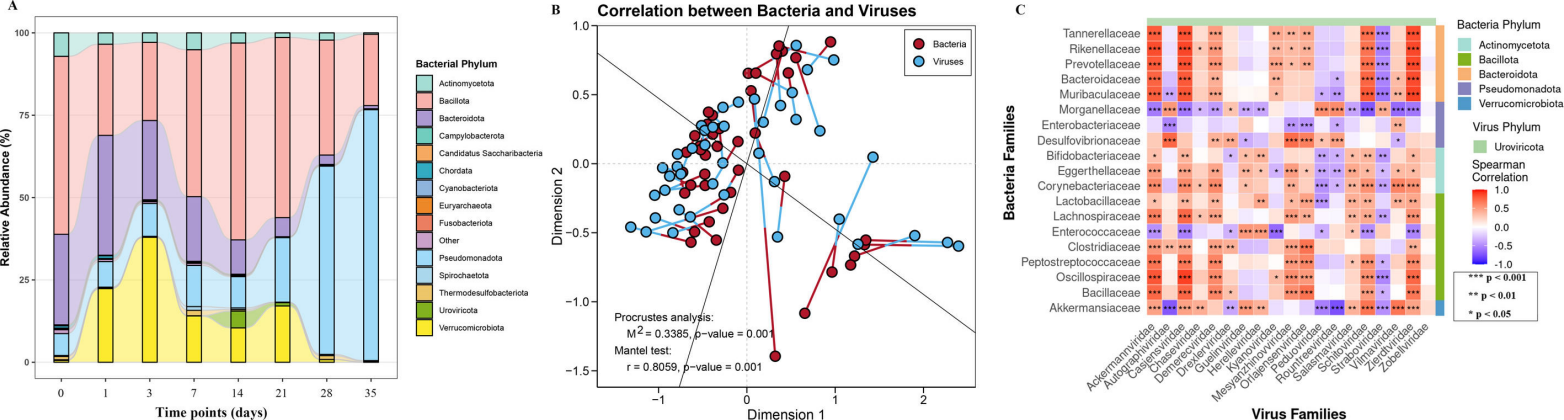
Bacterial-viral community dynamics and interactions. (**A**) Temporal dynamics of bacterial phylum-level relative abundance. (**B**) Procrustes analysis congruence between bacterial and viral communities (Mantel test: *r* = 0.8059, *P* = 0.001). (**C**) Spearman correlation between top bacterial families (representing five dominant phyla) and viral families.

During the early decomposition stage (0–3 days postmortem), rapid shifts occurred at the phylum level (Fig. 5A). Bacillota (formerly Firmicutes) dominated at Day 0 (54.0%), succeeded by Bacteroidota (formerly Bacteroidetes) as the predominant phylum at Day 1 (36.4%), with Verrucomicrobiota emerging as the most abundant phylum by Day 3 (38.0%). At the family level (Fig. S4), Lactobacillaceae and Akkermansiaceae were dominant. Correspondingly, the genera *Lactobacillus* and *Akkermansia* were dominant. The mid-stage (7–21 days) exhibited Bacillota re-establishing dominance (59.8% at Day 14) (Fig. 5A). At the family level, Enterococcaceae and Lactobacillaceae were dominant, while at the genus level, *Enterococcus* was predominant (Fig. S4). In the late stage (28–35 days), Pseudomonadota (formerly Proteobacteria) progressively increased from 19.6% at Day 21 to 76.2% at Day 35, supplanting Bacillota (which decreased from 54.7% to 21.6%). Family-level analysis showed Morganellaceae becoming the most abundant taxon. The genus *Proteus* were dominant (Fig. S4).

Alpha diversity analysis assessed bacterial diversity across various PMIs using the Shannon index, Richness index, and Simpson index. All indices showed a fluctuating decline throughout decomposition (Fig. S5A through C), indicating a reduction in bacterial diversity.

### Analysis of bacterial and viral community consistency and correlations during cadaver decomposition

Procrustes analysis (*M*² = 0.3385, *P* = 0.001) and Mantel tests (*r* = 0.8059, *P* = 0.001), both based on Bray-Curtis distance matrices, confirmed significant consistency between bacterial and viral communities (Fig. 5B), indicating synchronized ecological restructuring throughout decomposition.

A correlation analysis between the 19 most abundant bacterial families (spanning the phyla Bacteroidota, Bacillota, Pseudomonadota, Actinobacteriota, and Verrucomicrobiota) and 19 viral families at the family level (Fig. 5C) revealed significant interaction patterns. Specifically, five bacterial families within the phylum Bacteroidota exhibited significant positive correlations with the viral families Ackermannviridae, Casjensviridae, Zierdtviridae, Schitoviridae, and Demerecviridae, while showing a significant negative correlation with Straboviridae. Bacterial families in the phylum Bacillota also displayed significant correlations with the aforementioned five viral families (Ackermannviridae, etc.). The representative bacterial family Morganellaceae within the phylum Proteobacteria exhibited significant correlations with 16 out of the 19 analyzed viral families (excluding Kyanoviridae, Mesyanzhinovviridae, Orlajensenviridae, and Zobellviridae). Major bacterial families in the phylum Actinobacteriota showed correlations with the majority of the 19 viral families. Bacterial families in the phylum Verrucomicrobiota exhibited significant correlations with 14 viral families.

## DISCUSSION

Accurate estimation of the PMI is critically important in forensic science. Although bacterial and fungal successional patterns during decomposition have been extensively studied, viral dynamics—a key component of the microbiome—in buried cadavers remain systematically uncharacterized. This study presents systematic analysis of viral succession patterns in buried rat cadavers over a 35-day decomposition period. By integrating metagenomics with machine learning, we developed a predictive model demonstrating the potential utility of viruses as novel PMI biomarkers. Crucially, viruses exhibit environmental stability compared to bacteria due to protective protein capsids (33, 34), maintaining integrity under extreme conditions like high-altitude settings (35, 36)—thus enabling new PMI estimation possibilities in challenging environments. Furthermore, we discuss how viruses actively modulate bacterial communities through dynamic lysis-lysogeny transitions, promoting nutrient release and driving decomposition ecology.

During corpse decomposition, both viral and bacterial diversity declined with prolonged PMI. In the initial stages, organic compounds released from cadavers (e.g., amino acids, phospholipids) temporarily boost microbial growth. However, resource depletion leads to a decrease in nutrient-dependent microbial communities (9). Eutrophic conditions often favor fast-growing opportunists (e.g., Bacillota), suppressing rare taxa and reducing overall bacterial diversity (32). Viral diversity is inherently limited by the richness of bacterial hosts (37, 38); thus, reduced bacterial diversity constrains potential host range and diminishes viral diversity. Additionally, toxic metabolites (e.g., putrescine) may further inhibit microbial diversity.

Beta diversity analysis showed significant time-dependent changes in microbial communities during decomposition. Day 14 emerged as a critical ecological transition point. Early-stage communities exhibited high similarity and strong correlations (e.g., Days 0–3), reflecting homogeneity due to uniform microenvironments and nutrient availability. Day 14, both community similarity and correlation had declined sharply, indicating community structure reshaping. This may be due to resource exhaustion and environmental stressors that led to selective extinction of early-colonizing taxa while increasing phage-host conflicts reshaped community dynamics (39, 40). During late decomposition (Days 28–35), there was a resurgence in similarity (0.74) and near-perfect correlation (*r* = 0.99, *P* < 0.001). This indicated that the microbial community had entered a highly specialized and stable period. The surviving bacterial community and its phages had established a new balance through coevolution, and the strong correlation of the viral community highlights ecological resilience (i.e., the ability to maintain or restore structural and functional stability after disturbance).

Microorganisms are essential in cadaver decomposition, yet research has mainly focused on bacterial communities, while viruses—often called the “dark matter” of the microbiome—remain largely neglected. Metcalf et al. (41) analyzed bacterial succession on cadavers and created a PMI model; however, their findings apply only to indoor scenarios and do not translate well to buried cadaver environments. Zhang et al. (42) developed a RF model using rectal bacterial markers (29 OTUs), achieving a MAE of 2.06 days but did not separate training and testing sets. Similarly, Cui et al. (43) model based on grave soil microorganisms showed high precision with a low MAE of 1.27 days. However, this model also did not distinguish between training and testing sets. Moreover, its applicability in complex environments and long-term PMI scenarios is limited due to reliance on samples from a single soil source, which lacks coverage of regional microbial diversity. This study used the long-neglected viral community as core predictive markers. An ERT model, built with only 19 viral families, showed excellent performance on the test set (MAE = 2.54 days), demonstrating that viral succession has high temporal specificity. Key viral families exhibit significant abundance peaks at specific PMIs (e.g., during their host bacteria’s peak succession phase), providing precise temporal anchor points for the model. Bacteriophages actively influence their host bacterial communities through lysis (44); this bacteriophage-host interaction—rooted in specific co-evolutionary relationships—not only accelerates decomposition but also offers unique signals for tracing dynamics and inferring PMI, complementing traditional PMI estimation methods based on bacteria.

The ecological process of corpse decomposition centers on the dynamic succession of microbial communities. Bacteriophages, viruses specifically infecting bacteria, exhibit abundance and distribution closely tied to their hosts (17). Phages enhance bacterial diversity by exerting top-down control on high-abundance bacterial species (18), with bacteria and phages mutually influencing each other’s population dynamics (45). This study used Procrustes analysis and Mantel tests and confirmed a high coupling between viral and bacterial community dynamics during decomposition. Viral abundance showed significant correlations with key bacterial phyla, including Bacillota and Bacteroidota. Throughout this process, phages likely adapt their lytic and lysogenic infection strategies in response to changing nutrient conditions to maintain bacterial coexistence, potentially aligning with strategies like “Killing the Winner” (KtW), “Piggyback the Winner” (PtW), and “Piggyback the Loser” (PtL) (46–48). This suggests phages drive decomposition by regulating bacterial community structure. Notably, different phage strategies vary depending on environmental conditions (49).

During early decomposition (nutrient-rich phase), Bacillota dominate as the primary resource utilizers. During this phase, Peduoviridae abundance remains elevated, likely employing the KtW strategy—targeting Bacillota for lysis via recognition of surface molecules like lipopolysaccharides through tail fibers (50)—thereby preventing their resource monopoly. This process involves the “viral shunt” effect, releasing soluble nutrients (e.g., amino acids, nucleotides, phosphate compounds) through lysis (51). This not only creates ecological opportunities for disadvantaged groups like Pseudomonadota but also directly stimulates their proliferation (20, 52). These observations align well with the mechanism that “lytic strategies enrich the system via viral shunt under nutrient-rich conditions” and the KtW theory of “targeting dominant species to maintain diversity” (53, 54). Entering the middle decomposition stage (nutrient transition phase), nutrient levels gradually decline, and Bacillota abundance exhibits a transient rebound (day 14). The abundance pattern of Herelleviridae is consistent with the PtL strategy: it potentially remains latent via lysogeny at low host density and then switches to lytic infection as host density peaks, leading to a significant abundance increase. This density-dependent lysis likely controls host population size; the further release of nutrients via the viral shunt may drive bacterial community replacement (e.g., Pseudomonadota growth) while also helping stabilize the community by inhibiting excessive Bacillota expansion. This dynamic matches the PtL strategy’s switching mechanism (“lysogeny favored at low host density, lysis at high density”) and its property of “expanding coexistence range in fluctuating environments” (53). In the late decomposition stage (nutrient depletion phase), Pseudomonadota emerge as the new dominant species. Straboviridae may continue a PtL strategy, increasing in abundance with host density and suppressing excessive competition through lysis. In parallel, Peduoviridae, which target residual Bacillota, likely intensify the KtW strategy; this intensification is evidenced by a significant abundance increase. Nutrients released from this lysis may support system persistence under resource-scarce conditions. In summary, phages dynamically shift strategies across stages in response to changing resource conditions. They drive resource redistribution via lytic infection during nutrient-rich phases and stabilize community structure through lysogenic-lytic switching amid nutrient fluctuations. Ultimately, this facilitates the orderly succession of bacterial communities and ecosystem stability during corpse decomposition.

In summary, the dynamic successional changes and interactive mechanisms between viruses (particularly bacteriophages) and bacterial communities not only establish a novel foundation for viromics-based forensic PMI estimation but also elucidate how bacteriophages function as key ecological drivers in cadaver decomposition. Nevertheless, despite this study representing the first application of virome data to accurately estimate PMI in buried cadavers (MAE = 2.54 days), several limitations warrant consideration (9, 55, 56) and cadaver variability (e.g., body size, ante-mortem diet, and health status) (31) may significantly influence microbial/viral dynamics. These factors exacerbate system complexity by shaping individualized microbiome profiles—starkly contrasting with the stable gut microbiota observed in rat models under controlled dietary conditions. While rodent models provide a controlled framework for mechanistic investigation, extrapolating PMI estimation based on viral dynamics to human contexts remains challenging. Future research should prioritize systematic validation using human cadaver samples across diverse decomposition scenarios.

## MATERIALS AND METHODS

### Experimental design and sample collection

In this experiment, 48 rats were obtained from the Experimental Animal Centre of Shanxi Medical University and cohoused from birth. The rats were humanely euthanized by cervical dislocation. Six graves were prepared for each time point, and fecal samples associated with each cadaver were collected through destructive sampling. Fecal samples were collected from the rectum via dissection. Samples from six rats that were not buried served as day 0 controls. The remaining 42 rat cadavers were buried in individual soil graves at a depth of approximately 20 cm in an open area with homogeneous soil. The graves were covered with the excavated soil. The experiment spanned 35 days (from April 12 to May 17, 2024), with samples collected at eight time points: day 0, 1, 3, 7, 14, 21, 28, and 35. All experimental procedures complied with the ARRIVE guidelines and were conducted in accordance with the U.K. Animals (Scientific Procedures) Act, 1986, and associated guidelines. All collected samples were stored at −80°C until further processing.

### DNA extraction, library preparation, and metagenomic sequencing

Total DNA was extracted from fecal samples using the E.Z.N.A. Viral DNA Kit (Omega Bio-tek, Norcross, GA, USA) following the manufacturer’s instructions. DNA quality was assessed using spectrophotometry, and only high-quality samples (OD260/280 = 1.8–2.2, OD260/230 ≥ 2.0) were selected for downstream library preparation. Metagenomic libraries were constructed using the TruSeq Nano DNA Sample Preparation Kit (Illumina, San Diego, CA) with 1 µg of total DNA as input. The library preparation process included DNA end repair, A-tailing, and ligation of indexed adaptors, as described in the Illumina protocol. Libraries were size-selected for target fragments of approximately 400 bp using 2% Low Range Ultra Agarose gel electrophoresis and subsequently amplified by PCR using Phusion DNA polymerase (NEB) for 15 cycles. Sequencing was performed on an Illumina NovaSeq platform with a paired-end 150 bp (PE150) configuration by Shanghai Biozeron Biotechnology Co., Ltd. (Shanghai, China).

### Quality control, contig assembly, and taxonomic annotation

Raw paired-end reads were subjected to quality control and trimming using Trimmomatic (version 0.40) with the following parameters: SLIDINGWINDOW:4:15 and MINLEN:75 (57). Reads aligning to the mouse genome were removed to eliminate host contamination. The resulting high-quality reads were used for *de novo* assembly with MEGAHIT, employing the parameter “--min-contig-len 500” to generate contigs for each sample (58). Contigs longer than 2,000 bp were selected for viral identification using a combination of tools, including VirFinder (score > 0.7 and *P*-value < 0.05) (59), VirSorter2 (60), and the IMG/VR database (Integrated Microbial Genomes/Virus, https://img.jgi.doe.gov/cgi-bin/vr/main.cgi). Viral operational taxonomic units (vOTUs) were clustered using MUMmer software, with sequences sharing >95% similarity and >85% coverage considered part of the same vOTU. The longest sequence within each cluster was designated as the representative vOTU (61). PhaBox was used for taxonomic annotation of viral species taxonomic. For the analysis of bacterial data, taxonomic annotation was performed on quality-controlled and host-depleted reads using Kraken2 (62).

### Data analysis and visualization

Normalization was performed using the vegan package to calculate diversity indices, including species Shannon, Richness, and the Simpson diversity index. Visualizations were generated in R. Beta diversity analysis utilized the vegan package, while Spearman’s rank correlation coefficient (Spearman’s *r*) assessed correlations with statistical significance set at *P* < 0.05. Relationships between variables were visualized using the pheatmap package for correlation heatmaps. PCoA based on Bray-Curtis distance was conducted with the ape package’s pcoa function to visualize pairwise differences across time points (63). The Bray-Curtis distance matrix and community similarity (calculated as 1—Bray-Curtis distance) were computed using vegdist from the vegan package (64). Additionally, PERMANOVA evaluated PMI’s effect on viral community composition in burial cadavers. Procrustes analysis was used to examine the congruence between bacterial and viral communities, while Mantel and Spearman tests were employed to evaluate the correlations between them. Data preprocessing involved Hellinger standardization, followed by the calculation of Bray-Curtis distance matrices using the vegdist function from the vegan package. Mantel tests were performed using the mantel function in the same package to quantify correlations between distance matrices.

Statistical analyses were performed in R, with the caret package used to partition data into training and testing sets (2:1 ratio) (65). All data were preprocessed through Hellinger standardization before model construction. We developed and compared multiple predictive models, including linear regression (stats package) (66), random forest (randomForest package) (67), support vector machine (e1071 package) (68), Extra Trees (ranger package) (69), and Lasso/Elastic Net regression (glmnet package) (70). The parameter settings and optimization methods related to the above-mentioned model are detailed in Table S2. To enhance model interpretability, we employed SHAP values from the iml package, which quantify feature importance by determining each predictor’s marginal contribution to individual predictions, thereby providing insights into model decision-making processes.

## Data Availability

The raw metagenomic sequencing data generated in this study have been deposited in the NCBI Sequence Read Archive (SRA) and are publicly available under BioProject accession number PRJNA1293747.

## SUPPLEMENTAL MATERIAL

Supplemental material aem.01453-25-s0001.docx Figures S1 to S5; Tables S1 and S2.

ASM does not own the copyrights to Supplemental Material that may be linked to, or accessed through, an article. The authors have granted ASM a non-exclusive, world-wide license to publish the Supplemental Material files. Please contact the corresponding author directly for reuse.
