# Supplementary material for: Viral community succession during cadaver decomposition and its potential for estimating postmortem intervals
Source: Appl Environ Microbiol. 2025 Sep 16;91(10):e01453-25. doi: 10.1128/aem.01453-25 (PMC12542767; doi:10.1128/aem.01453-25)
Supplement: Supplemental material — Figures S1 to S5; Tables S1 and S2. [file aem.01453-25-s0001.docx]

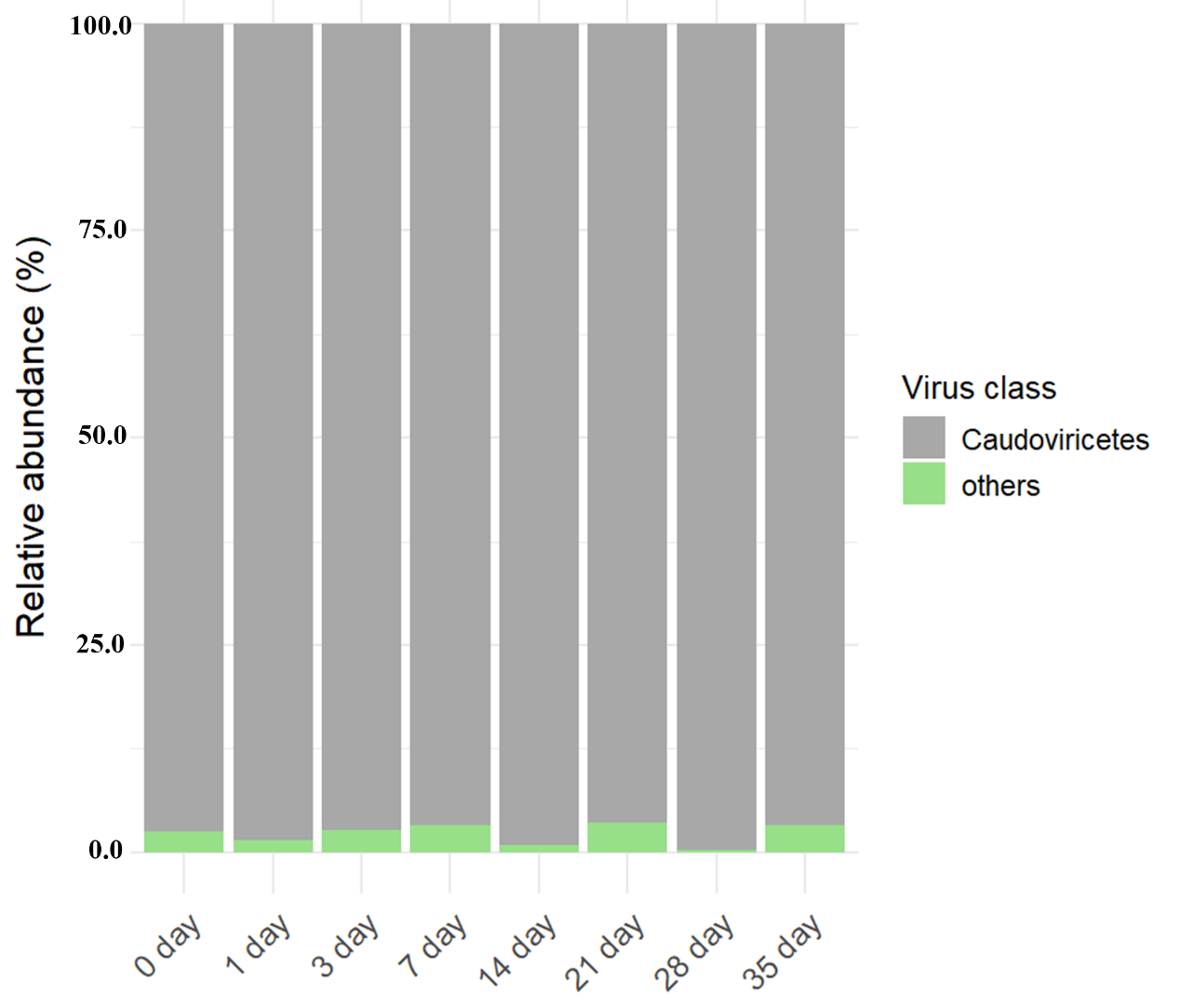


Figure S1. Relative abundance (%) of viral classes over time. X-axis: time points (days) during decomposition of buried cadavers.


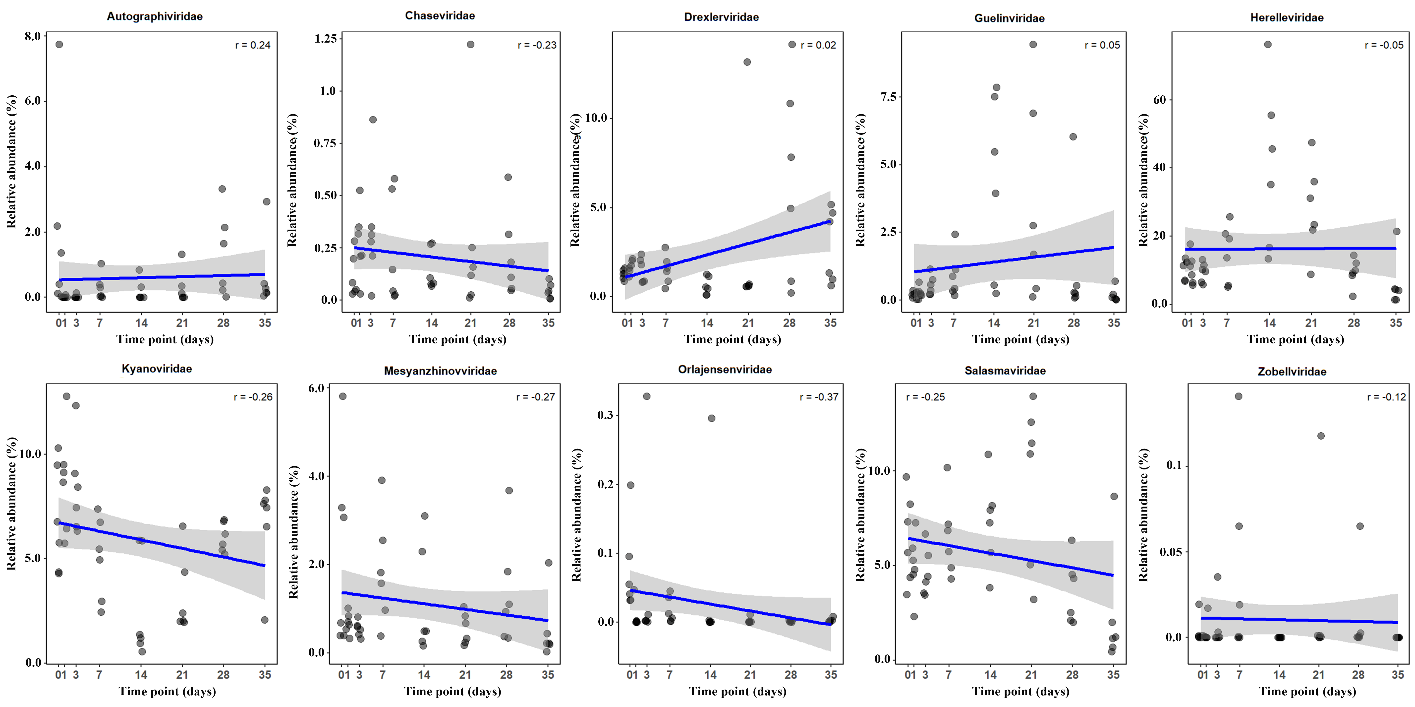


Figure S2. Correlation between virus family dynamics and PMI. The scatter plot shows that the time abundance of the selected virus family in the decomposition stage has no significant correlation with PMI.


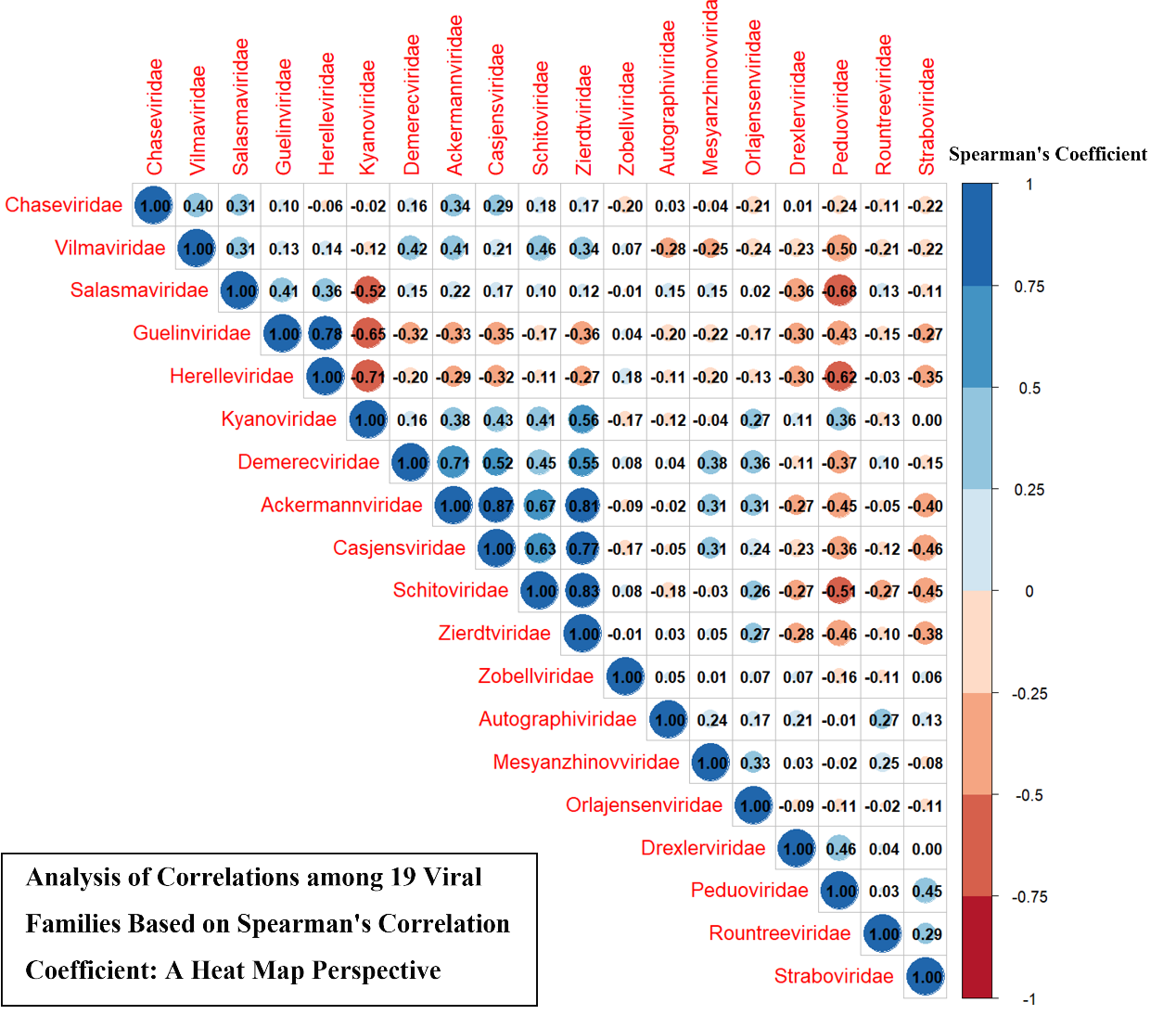


Figure S3. The correlation between viruses detected in the process of decomposition is further evaluated.


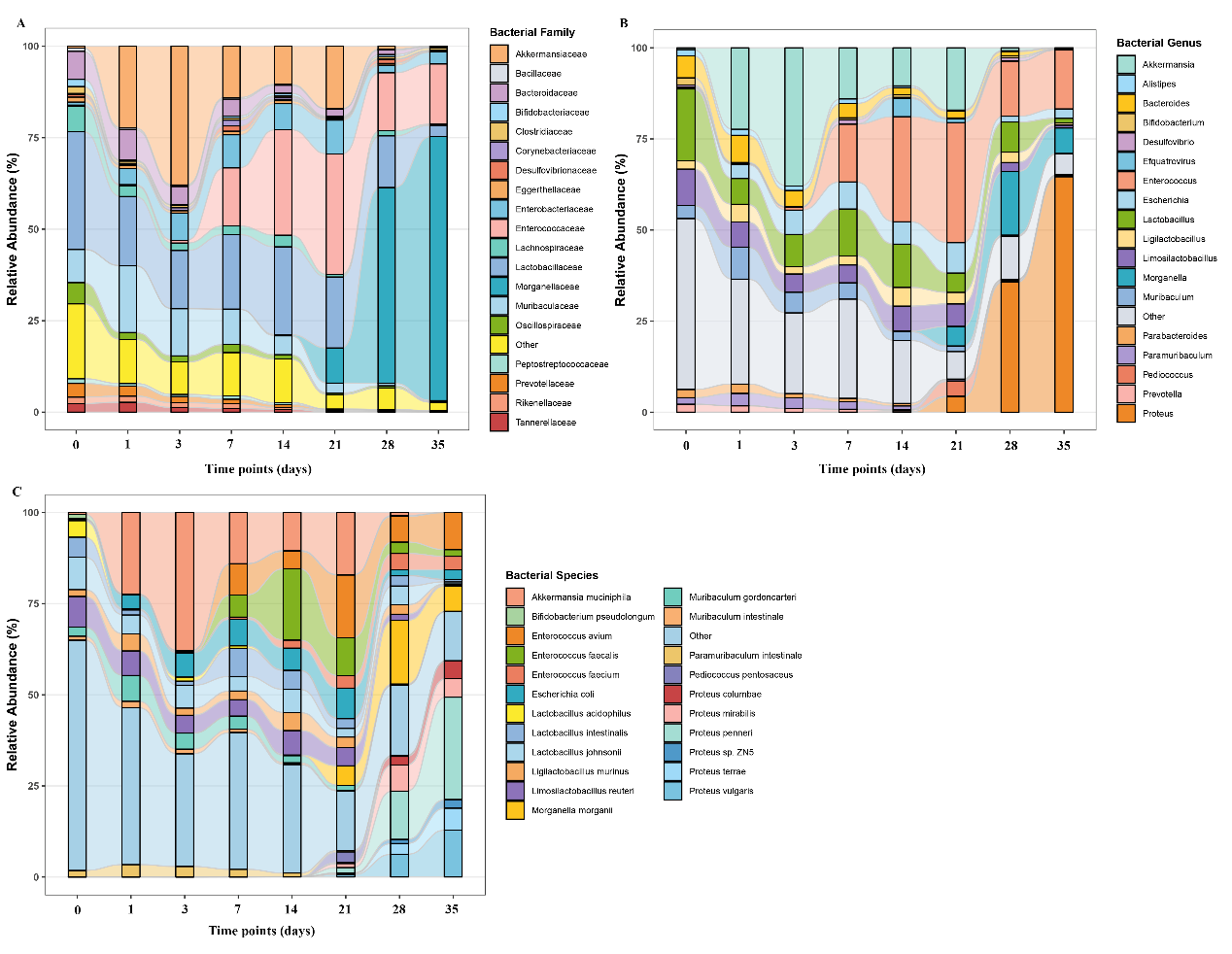


Figure S4. Relative abundance (%) of bacterial families/genus/species over time. X-axis: time points (days) during decomposition of buried cadavers.


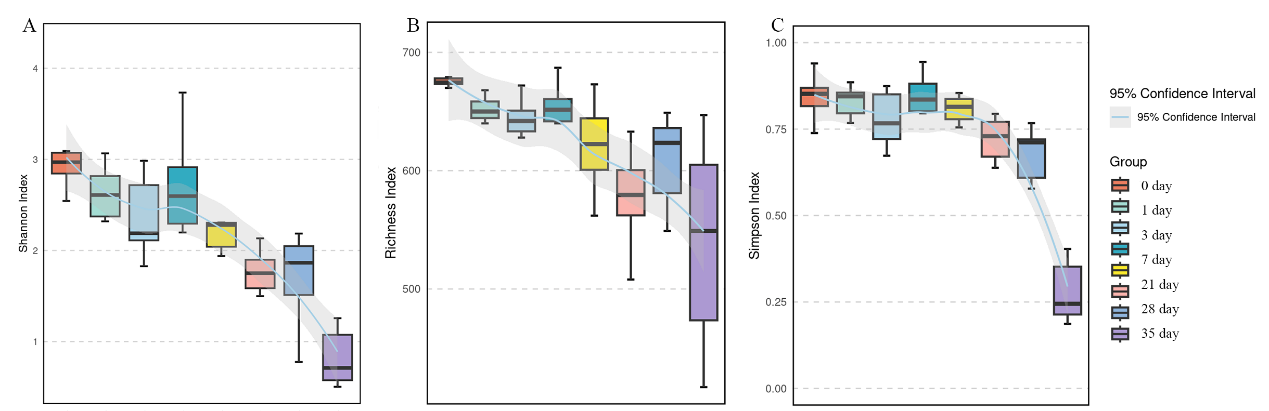


Figure S5. Bacterial community diversity during buried cadaver decomposition.

Alpha diversity indices: (A) Shannon, (B) Richness, and (C) Simpson at different time points.

Table S1 PERMANOVA testing the effects of PMI on beta- diversity.

| Factors | Df | SS | F | R^2^ | P | Significance |
| --- | --- | --- | --- | --- | --- | --- |
| PMI | 1 | 0.99 | 17.01 | 0.27 | 0.001 | *** |
| Residual | 46 | 2.68 |  | 0.73 |  |  |
| Total | 47 | 3.67 |  | 1 |  |  |

Table S2 Hyperparameter Optimization Framework

| Model | Core Parameter Settings | Optimization Method |
| --- | --- | --- |
| ERT | mtry = ncol(train_X)/2 (half features per split), **splitrule = "random"**, **ntree = 500** | Automatic feature selection via variable importance; no manual tuning. |
| ENR | α **∈ [0, 1]** (L1/L2 regularization mix), **λ ∈ [0.001, 1]** | 5-fold CV (**cv.glmnet**) for optimal α and λ. |
| LASSO | α = 1 (pure L1), **λ ∈ [0.001, 1]** | 5-fold CV for λ; feature screening via coefficient sparsity. |
| RF | ntree = 500, **importance = TRUE** | Implicit optimization via Out-of-Bag (OOB) error. |
| SVR | kernel = "radial", **C ∈ [0.1, 100]**, **γ ∈ [0.001, 10]** | Grid search + 5-fold CV (10× step size) for kernel/regularization parameters. |
| LR | No additional parameters (OLS fitting) | Baseline model for performance comparison. |
